# Supplementary figures and images for: Different Innate and Adaptive Immune Responses to SARS-CoV-2 Infection of Asymptomatic, Mild, and Severe Cases
Source: Front Immunol. 2020 Dec 16;11:610300. doi: 10.3389/fimmu.2020.610300 (PMC7772470; doi:10.3389/fimmu.2020.610300)

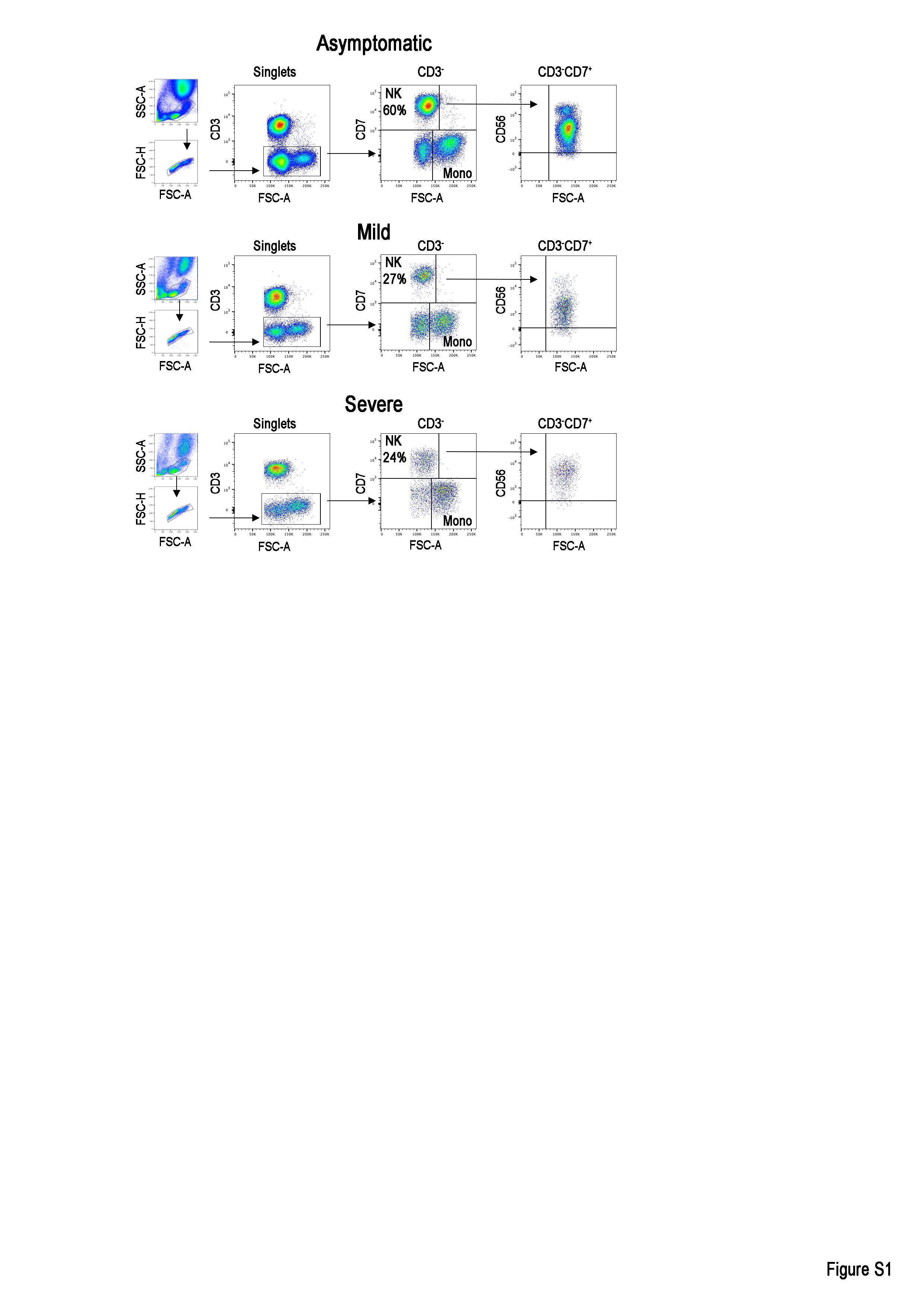

Supplement: Supplementary Figure 1 — The lympho-monocyte gate was designed based on physical characteristics (FSC-A vs SSC-A). Singlets are identified by FSC-H vs FSC-A parameters. NK were identified as CD3-CD7+FSC-Alow and monocytes as CD3-CD7-FSC-Ahigh. NK cells identified as CD7+ in the CD3- gate also express CD56. [file Image_1.tif]

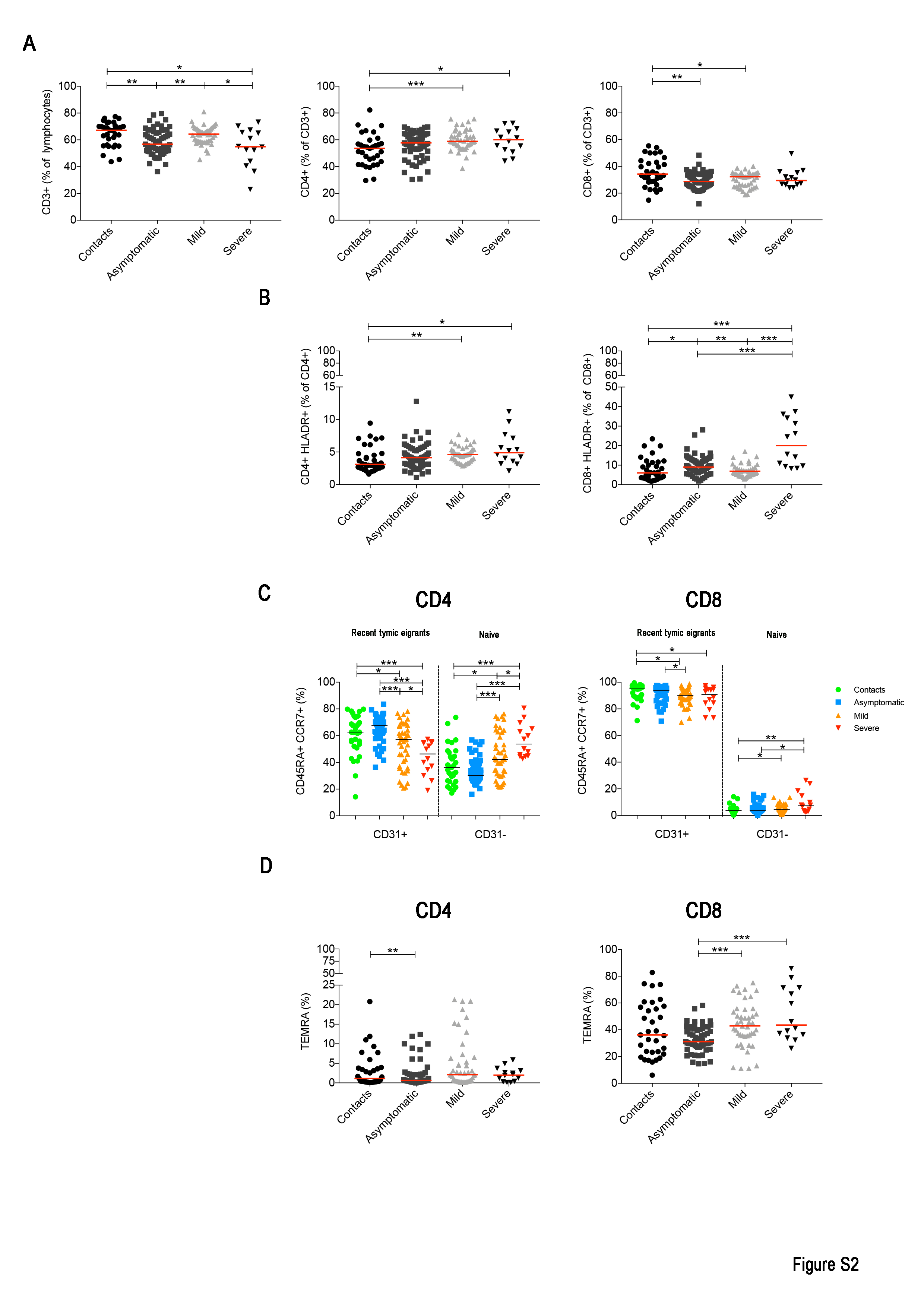

Supplement: Supplementary Figure 2 — (A) Scatter plots show percentage of T cells (CD3+), CD4+ and CD8+. (B) Plots show the percentage of CD4+HLADR+and CD8+HLADR+ T cells in all sample serially collected and analyzed in the study. (C) Naïve T cells were divided based on CD31 expression (CD31+ and CD31-). (D) Plots show the percentage of TEMRA in CD4+ and CD8+ T cells in all sample serially collected and analyzed in the study. Midlines indicate median. Statistical significances were determined using unpaired, two-tailed Mann-Whitney U-tests. *p ≤ 0.05, **p < 0.01, ***p < 0.001. [file Image_2.tif]

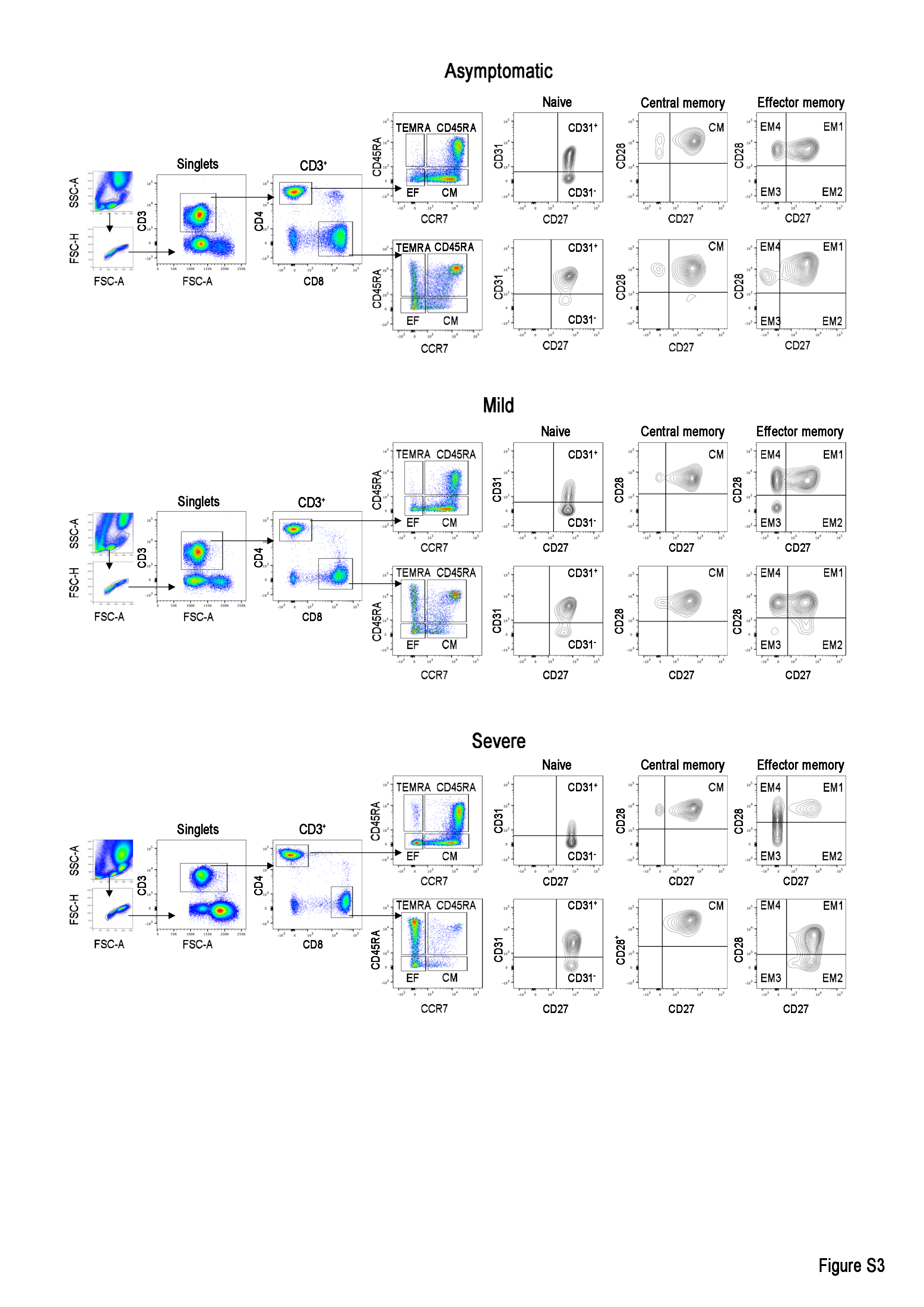

Supplement: Supplementary Figure 3 — Gating strategy to analyze T cells subset. The CD4+ and CD8+ T cells were subdivided into the main T cell subsets. HLADR was used to identify activated CD4+ and CD8+ T cells. Using CD45RA and CCR7: naïve (CD45RA+CCR7+), central memory (CM CD45RA-CCR7+), effector memory (EM CD45RA-CCR7−) and TEMRA (CD45RA+CCR7−) T cells were identified. Naïve T cells were further divided based on CD31 expression (CD31+ and CD31−). CM and EM were separated based on the expression of CD27 and CD28. [file Image_3.tif]

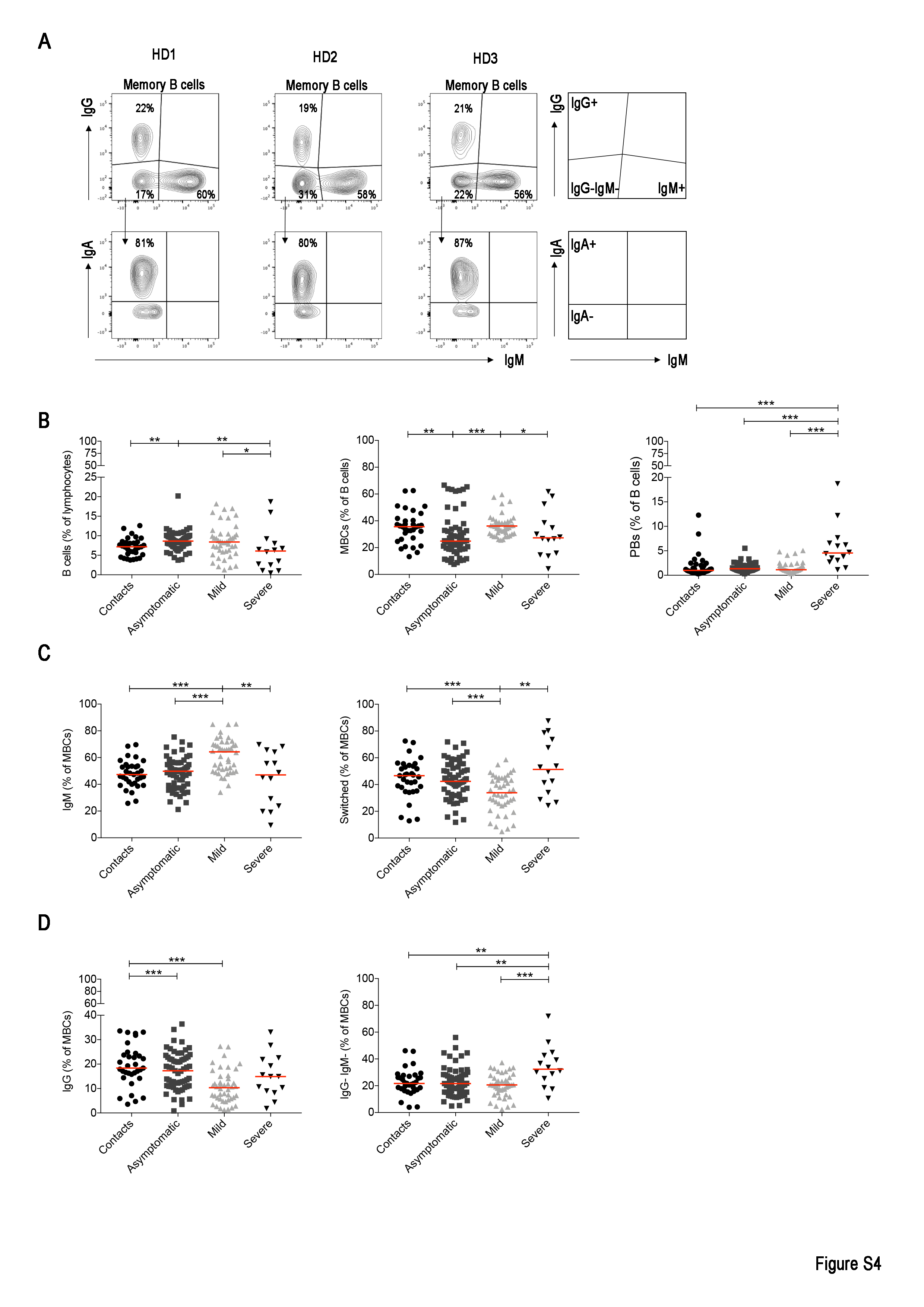

Supplement: Supplementary Figure 4 — (A) FACS plots in three healthy donors indicates that most of IgG-IgM- MBCs correspond to IgA expressing MBCs and minimal part of these cells are IgA-IgG−IgM− MBCs. (B) Plots indicate the percentage of B cells, MBCs and plasmablasts. In (C) we show the frequencies of IgM and switched MBCs. In (D) we show the frequency of IgG+, IgG−IgM−MBCs. (B–D) Graphs refer to all samples analyzed in the study. Midlines indicate median. Statistical significances were determined using unpaired, two-tailed Mann–Whitney U-tests. *p ≤ 0.05, **p < 0.01, ***p < 0.001. [file Image_4.tif]
