# Supplementary material for: Different Innate and Adaptive Immune Responses to SARS-CoV-2 Infection of Asymptomatic, Mild, and Severe Cases
Source: Front Immunol. 2020 Dec 16;11:610300. doi: 10.3389/fimmu.2020.610300 (PMC7772470; doi:10.3389/fimmu.2020.610300)
Supplement: Supplementary file 5 [file DataSheet_1.pdf]

Supplementary Table 1: Characteristics of the study population

A

Study Group	COVID-19 severe	COVID-19 mild	SARS-Cov-2 asymptomatic	SARS-Cov-2 contacts
number of patients	8	8	20	28
age, median (IQRs)	66 (52-82.5)	54 (48-63.5)	37 (34.3-46.8)	36.5 (32-48.5)
sex (M/F)	6/2	5/3	4/16	8/20

B

Study Group	COVID-19 ICU	COVID-19 non-ICU
number of patients	34	43
age, median (IQRs)	62 (55-73.25)	69 (47-76)
Day of analysis (from admission, median (IQRs))	2 (0.75-4.00)	2 (1-3)

Supplementary Table 2: Antibodies panel

	Antibodies	Source	Identifier
T cell panel	CD45RA (Clone HI100) FITC	BD Biosciences	555488
	CCR7 (Clone CD197) PE	BD Pharmingen	566741
	CD3 (Clone SK7) PerCP	BD Biosciences	345766
	CD4 (Clone RPA-T4) Pe-Cy7	Immunological Sciences	MAB-19522PC7
	CD27 (Clone M-T271) APC	BD Pharmingen	558664
	CD8 (Clone SK1) APC-Cy7	BD Biosciences	348793
	CD28 (Clone CD28.2) BV421	BD Horizon	562613
	CD31 (Clone WM59) BV605	BD Horizon	562855
B cell panel	CD45 (Clone 2D1) V500	BD Biosciences B-cell tube	626220
	CD19 (Clone SJ25C1) PE-Cy7		
	CD24 (Clone ML5) PE		
	CD27 (Clone L128) APC		
	CD38 (Clone HIT2) PercP-Cy5.5		
	IgM (Clone G20-127) FITC		
	IgG (Clone G18-145) APC-H7		
	IgD (Clone IA6-2) V450		
	CD21 (Clone B-ly4) BV605	BD OptiBuild	740395
Monocyte panel	CD45 (Clone 2D1) V500	BD Biosciences	655873
	CD3 (Clone UCHT1) BV605	BD OptiBuild	742623
	CD14 (Clone M5E2) PE	BD Pharmingen	555398
	CD16 (Clone LNK16) FITC	Immunological Sciences	MAB-1810F
	CD4 (Clone RPA-T4) PE-Cy7	Immunological Sciences	MAB-19522PC7
	CD8 (Clone SK1) APC-H7	BD Biosciences	641400
	CD38 (Clone HIT2) PerCP	Immunological Sciences	MAB-938PCP
	HLA-DR (Clone L243) V450	BD Biosciences	655874
	CD33 (Clone P67.6) APC	BD Biosciences	345800
NK cell panel	CD45 (Clone 2D1) V500	BD Biosciences	655873
	CD3 (Clone SK7) PerCP	BD Biosciences	345766
	CD4 (Clone RPA-T4) PE-Cy7	Immunological Sciences	MAB-19522PC7

	CD8 (Clone SK1) APC-H7	BD Biosciences	641400
	CD7 (Clone MEM-92) APC	Immunological Sciences	MAB-1006A
	CD56 (Clone MY31) PE	BD Biosciences	345810
	CD16 (Clone LNK16) FITC	Immunological Sciences	MAB-1810F
**Figure S7 staining**	CD19 (Clone SJ25C1) BUV737	BD Biosciences	Cat#612757
	CD24 (Clone ML5) BV711	BD Biosciences	Cat#563450
	CD27 (Clone M-T271) PE	BD Biosciences	Cat#557330
	CD38 (Clone HIT2) BV421	BD Biosciences	Cat#562445
	IgM (Polyclonal) Alexa Fluor 647	Jackson Immunoresearch	Cat#109-606-129
	IgD (Clone IA6-2) FITC	BD Biosciences	Cat#562023
	IgG (Clone G18-145) BUV395	BD Biosciences	Cat#564229
	IgA (Clone IS11-8E10) FITC	Miltenyi Biotec	Cat#130-113-475

Supplementary Table 3: Monocytes mean and median values

Patients group	Contacts	Asymptomatic	Mild	Severe
	mean ± SD<br>median/IQR	mean ± SD<br>median/IQR	mean ± SD<br>median/IQR	mean ± SD<br>median/IQR
Monocytes<br>(% of lymphocytes)	13.1 ± 6.1<br>11.9/8.2-17.8	13.3 ± 5.8<br>14.9/8.2-17.7	17.5 ± 6.2<br>15.6/12.9-20	37.1 ± 19.2<br>33/23-54.1
Classical<br>(% monocytes)	55.1 ± 12.8<br>53.5/42.9-67.2	60.6 ± 10.2<br>62.1/54.6-67.9	57.8 ± 12.8<br>58.1/47.9-68.6	61.8 ± 19.8<br>70.3/46.2-76
Intermediate<br>(% monocytes)	14.8 ± 8.5<br>13.6/7.6-23.1	13 ± 8.2<br>12.6/4.9-22	13.6 ± 10.6<br>8.9/7.8-24.3	23.3 ± 14.8<br>20.9/13.3-29.5
Non Classical<br>(% monocytes)	12.1 ± 6.2<br>13.2/7.3-16.7	10.4 ± 5.4<br>11.4/4.8-14.2	13.1 ± 4.8<br>11.1/9.9-18.6	5 ± 5.7<br>3.1/0.6-10.1

Supplementary Table 4: Monocytes total mean and median values

Patients group	Contacts	Asymptomatic	Mild	Severe
	mean ± SD<br>median/IQR	mean ± SD<br>median/IQR	mean ± SD<br>median/IQR	mean ± SD<br>median/IQR
Monocytes<br>(% of lymphocytes)	13.2 ± 5.1<br>11.8/10.2-16.4	15.1 ± 5.5<br>14.9/10.7-19.4	16.2 ± 4.3<br>16/14.1-18.2	29.8 ± 16<br>25.1/19-36.1
Classical<br>(% monocytes)	48 ± 21.1<br>52.8/35.4-64	58.9 ± 16<br>62.9/50.1-72.3	61.9 ± 12.3<br>65.4/56.3-70.9	65.1 ± 19<br>71.8/44.7-77.3
Intermediate<br>(% monocytes)	26.9 ± 17.5<br>22.4/14-35.4	20.3 ± 13.6<br>16.7/10.4-26.4	16.4 ± 11.3<br>13.2/9.7-15.6	21.8 ± 15.7<br>16.9/10.8-37.3
Non Classical<br>(% monocytes)	7.1 ± 4.9<br>6.2/3.4-9.5	6.1 ± 3.7<br>5.9/3.5-7.8	6 ± 3.5<br>5.2/3.8-7.9	1.7 ± 2.4<br>0.3/0.1-3.4

Supplementary Table 5: NK cells mean and median values

Patients group	Contacts	Asymptomatic	Mild	Severe
	mean ± SD<br>median/IQR	mean ± SD<br>median/IQR	mean ± SD<br>median/IQR	mean ± SD<br>median/IQR
NK<br>(% of CD3-)	38.9 ± 14.2<br>36.5/29.2-47.8	38.8 ± 10.7<br>39.4/30.6-48.3	29 ± 9.3<br>27.8/22.5-38.9	14.6 ± 13.1<br>6.7/4.2-29.5
Monocytes<br>(% of CD3-)	30.3 ± 9.9<br>29.7/23-38.6	28.5 ± 8.5<br>31.1/18.6-33.9	39.5 ± 8.5<br>42.7/33-46	62.3 ± 20.1<br>61/42.8-82
Monocytes/NK<br>ratio	0.9 ± 0.6<br>0.9/0.5-1.2	0.8 ± 0.4<br>0.8/0.4-1	1.5 ± 0.6<br>1.4/1-1.9	10.6 ± 10.3<br>8.4/1.9-17.4
Monocytes/Lymphocytes<br>ratio	0.2 ± 0.09<br>0.1/0.09-0.2	0.2 ± 0.08<br>0.2/0.09-0.2	0.2 ± 0.1<br>0.2/0.1-0.3	0.8 ± 0.8<br>0.5/0.3-1.2

Supplementary Table 6: NK cells total mean and median values

Patients group	Contacts	Asymptomatic	Mild	Severe
	mean ± SD<br>median/IQR	mean ± SD<br>median/IQR	mean ± SD<br>median/IQR	mean ± SD<br>median/IQR
NK<br>(% of CD3-)	38.3 ± 13.5	41 ± 10.5	29.3 ± 11.1	17.5 ± 12.7
Monocytes<br>(% of CD3-)	32 ± 9.2<br>31/25.8-38.2	31.1 ± 8.7<br>32.2/24.6-38	39 ± 10.2<br>39.8/31.2-48	60.7 ± 17.1<br>57.2/47.8-70.4
Monocytes/NK<br>ratio	1 ± 0.5<br>1.1/0.6-1.4	0.9 ± 0.5<br>0.8/0.5-1.1	1.6 ± 0.8<br>1.5/1-2.1	9.6 ± 11.7<br>3.8/2.3-14.4

Supplementary Table 7: T cells mean and median values

Patients group	Contacts	Asymptomatic	Mild	Severe
	mean ± SD<br>median/IQR	mean ± SD<br>median/IQR	mean ± SD<br>median/IQR	mean ± SD<br>median/IQR
CD3+<br>(% of lymphocytes)	70 ± 8.3<br>71.9/66-75.5	67.2 ± 8.1<br>66/62-71.2	69.2 ± 7.9<br>72/61.9-75.6	55.4 ± 16.1<br>56.7/43.1-70.4
CD4+<br>(% of CD3+)	60 ± 11.7<br>59.9/53.1-70.1	62.3 ± 7.8<br>62/56.6-68.6	64.4 ± 8.8<br>62.2/56.9-74.4	66.4 ± 10.9<br>71.8/54.7-73.6
CD4+ HLADR<br>(% of CD4+)	5.3 ± 2.4<br>4.8/3.8-6.5	6.9 ± 3.9<br>6.6/3.9-9.5	7.1 ± 1.8<br>6.5/5.8-8.6	9.5 ± 3.4<br>8.8/6.6-12.6
CD4+ central memory<br>(% of CD4+)	42.7 ± 7.5<br>42/35.4-49.2	46.9 ± 8.9<br>48.6/41.7-52.7	50.3 ± 5.4<br>51.7/45.5-54	42.7 ± 12.3<br>42.9/34-51.5
CD4+ effector memory<br>(% of CD4+)	13.4 ± 6.2<br>13.8/8.7-16.2	14.2 ± 6.5<br>14.5/9.9-18.1	11.7 ± 4.1<br>13.2/13.8-9.2	13.3 ± 6.1<br>14.8/6.8-18.2
CD4+ effector memory 1<br>(% of CD4+ effector memory)	61.4 ± 14.5<br>59.9/49.3-75.6	59 ± 13.4<br>61.5/54.8-67	55.7 ± 14.8<br>59.3/50.3-64.7	54.6 ± 16.3<br>52.8/46.2-66.3
CD4+ effector memory 2<br>(% of CD4+ effector memory)	0.9 ± 2<br>04./0.1-0.7	2.1 ± 2.4<br>1.4/0.4-2.9	0.5 ± 0.6<br>0.2/0.1-0.9	0.5 ± 0.5<br>0.3/0.2-0.7
CD4+ effector memory 3<br>(% of CD4+ effector memory)	9.1 ± 9.5<br>5.7/1.2-13.7	11.7 ± 16.8<br>6.9/0.7-14.9	10.7 ± 12.9<br>7.9/1.2-13.6	14.4 ± 10.7<br>14/4.3-25.8
CD4+ effector memory 4<br>(% of CD4+ effector memory)	28.7 ± 9<br>26.5/21.3-38.5	27.2 ± 6.3<br>27.9/23.2-31	33.1 ± 6.3<br>32.5/30.3-35	30.5 ± 14.2<br>28/18.5-41
CCR7+ CD45RA+<br>(% of CD4+)	39.3 ± 11.3<br>38.8/33-45.5	34.2 ± 10.6<br>35.5/27.9-39.6	31.9 ± 6.6<br>29.5/26.4-36.9	39.3 ± 16.7<br>35.8/26.1-51.4
CCR7+ CD45RA+ CD31-<br>(% of CD4+ naive)	35.7 ± 12.6<br>36/25.3-43.7	34.9 ± 10.3<br>33.7/27.9-40.2	48.1 ± 17.1<br>46.9/34.5-62	57.3 ± 14.5<br>54.4/44.4-71.8
CCR7+ CD45RA+ CD31+<br>(% of CD4+ CCR7+ CD45RA+)	62.9 ± 13.1<br>62.8/55.4-74.4	63 ± 11.4<br>64.8/56.5-70	46.6 ± 14.6<br>49.2/35-55.5	42.4 ± 14.5<br>45.4/28.1-54.6
CD4+ TEMRA<br>(% of CD4+)	1.7 ± 2.4<br>0.6/0.2-2.4	2 ± 3.5<br>0.5/0.2-1.7	2.7 ± 3.1<br>1.2/0.2-5.2	1.7 ± 1.8<br>1.4/0.2-2.7
CD8+<br>(% of CD3+)	31.6 ± 10.3<br>30.8/23.3-38.6	28.9 ± 7.2<br>27.4/24-34.2	28.9 ± 6.6<br>29.6/22.2-35.3	27.8 ± 9.3<br>24.4/22.8-34.5
CD8+ HLADR<br>(% of CD8+)	14 ± 9.8<br>11.2/7.5-20.4	19.5 ± 12.1<br>16.1/10.9-27.1	14.3 ± 5.9<br>14.3/9.2-20.4	45.1 ± 17.5<br>44.7/27.5-63.2
CD8+ central memory<br>(% of CD8+)	7.7 ± 4.7<br>6.6/4.4-11.3	10.8 ± 5.2<br>10.3/7.8-12.6	11.8 ± 6.2<br>10.9/7.3-12.8	7.5 ± 5.1<br>6.7/2.8-12
CD8+ effector memory<br>(% of CD8+)	14.7 ± 7.1<br>13.4/9-19	19.6 ± 8.7<br>19.1/13.8-23	13.7 ± 6.2<br>12.4/9.1-20.4	17.5 ± 10.1<br>18.7/6.8-27.7
CD8+ effector memory 1<br>(% of CD8+ effector memory)	67 ± 13.7<br>67.7/60.7-76	62.8 ± 14.8<br>66.2/55.2-74.4	66.2 ± 11.4<br>65.8/55.2-78.2	56.7 ± 21.6<br>57.1/38.1-76.7
CD8+ effector memory 2<br>(% of CD8+ effector memory)	13.2 ± 8.8<br>9.8/6.2-21.7	15.8 ± 10.1<br>15.2/8.2-25.2	11.2 ± 9.9<br>8.7/4.8-13.3	34.6 ± 23.5<br>36.8/13.1-47.9
CD8+ effector memory 3<br>(% of CD8+ effector memory)	10.1 ± 9.4<br>7.2/3.3-13.8	12.2 ± 14.1<br>9.7/4.1-12.6	9.2 ± 7.3<br>6.4/3.2-15.8	5.9 ± 5.8<br>4/2-11.9
CD8+ effector memory 4<br>(% of CD8+ effector memory)	9.7 ± 5.8<br>8.6/5-15.3	9.2 ± 5.1<br>7.9/6-10.6	13.4 ± 5.8<br>12.1/8-18.7	2.7 ± 2.6<br>1.8/0.5-5.5
CCR7+ CD45RA+<br>(% of CD8+)	35.8 ± 15.5<br>36.4/22.9-48.4	35.3 ± 14.7<br>39/19.9-43.3	29.9 ± 8.4<br>30.7/21.5-38.2	16.8 ± 8.5<br>17.4/9.1-23.5
CCR7+ CD45RA+ CD31-<br>(% of CD8+ CCR7+ CD45RA+)	5.3 ± 3.2<br>5.1/3.8-5.9	5.8 ± 4.4<br>4.8/3.3-6.2	6.6 ± 2.8<br>6.4/4.9-7.9	12.4 ± 9.1<br>10.3/4.2-19
CCR7+ CD45RA+ CD31+<br>(% of CD8+ CCR7+ CD45RA+)	90.4 ± 5.6<br>93.1/85.1-94.1	89.2 ± 6.3<br>90/87-93.6	79.2 ± 10.5<br>80.8/68-88.9	85.3 ± 9<br>87.3/78.3-92.2
CD8+ TEMRA<br>(% of CD8+)	37.5 ± 20.2<br>30.8/20.6-57.2	29.7 ± 12.3<br>28.8/21-40.2	40 ± 14.3<br>45.5/31.3-51	55.2 ± 22.7<br>52.6/36-76.9

Supplementary Table 8: T cells total mean and median values

Patients group	Contacts	Asymptomatic	Mild	Severe
	mean ± SD<br>median/IQR	mean ± SD<br>median/IQR	mean ± SD<br>median/IQR	mean ± SD<br>median/IQR
CD3+<br>(% of lymphocytes)	70.3 ± 7.8<br>71.9/68.1-75.2	64 ± 8.3<br>63.3/59.1-69.1	72 ± 6.1<br>73.2/67.8-76.6	62 ± 16.3<br>66.6/53.6-74.2
CD4+<br>(% of CD3+)	56.9 ± 11.8<br>58.1/47.4-64.2	61 ± 8.4<br>61.8/56.4-68.1	64.2 ± 7.2<br>62.3/58.7-70.6	64.9 ± 9.2<br>68.3/56.2-73.3
CD4+ HLADR<br>(% of CD4+)	4 ± 1.9<br>3.1/2.6-4.8	4.4 ± 1.9<br>4.1/3.1-5.6	4.7 ± 1.1<br>4.6/3.8-5.4	5.5 ± 2.6<br>4.9/3.5-7.3
CD4+ central memory<br>(% of CD4+)	34.3 ± 8.5<br>35.6/29.9-41.2	41.2 ± 7.5<br>40.9/37.3-46.4	38.9 ± 7.6<br>38.3/33.9-44.3	42.7 ± 10.4
CD4+ effector memory<br>(% of CD4+)	18.2 ± 8<br>17.7/13.2-23.9	19.7 ± 6.9<br>21.1/13.9-24.1	20.4 ± 7.9<br>20.1/14.6-25.7	17.9 ± 8.7<br>15.4/11.9-24.1
CD4+ effector memory 1<br>(% of CD4+ effector memory)	63.2 ± 13.5<br>62.3/54.9-75.2	64.9 ± 10.5<br>66.7/60.4-73.2	56.3 ± 18.7<br>63.3/54.4-67.6	61.4 ± 17.2<br>65.4/51.1-75.6
CD4+ effector memory 2<br>(% of CD4+ effector memory)	0.5 ± 0.9<br>0.3/0.1-0.6	0.7 ± 1.1<br>0.2/0.1-0.6	0.6 ± 1<br>0.2/0.07-0.6	0.5 ± 0.4<br>0.5/0.2-1
CD4+ effector memory 3<br>(% of CD4+ effector memory)	7.8 ± 9.3<br>3.6/0.7-12.1	7.3 ± 10.2<br>4.2/0.3-9.8	11.1 ± 14.3<br>5.3/3.3-9.3	9.1 ± 7.5<br>7.7/2.3-16.4
CD4+ effector memory 4<br>(% of CD4+ effector memory)	28.5 ± 8.2<br>26.3/22.4-36.8	27.2 ± 5.1<br>26.3/23.7-31.2	32 ± 6.7<br>31.3/27.8-35.4	29 ± 14.2<br>23.6/19.8-34.5
CCR7+ CD45RA+<br>(% of CD4+)	41.4 ± 11.9<br>40.3/34.1-50.6	34.2 ± 11.2<br>35.8/24.8-42.8	32.6 ± 9.1<br>34.9/25.6-40.4	40.3 ± 14.7<br>36.3/28.1-52
CCR7+ CD45RA+ CD31-<br>(% of CD4+ naive)	37.6 ± 14<br>36.2/26-46	33.2 ± 9<br>30.3/26.6-38	45.4 ± 16.3<br>42.1/32.3-60.8	56.3 ± 12.5<br>53.7/44.7-66.2
CCR7+ CD45RA+ CD31+<br>(% of CD4+ CCR7+ CD45RA+)	60.8 ± 15.3<br>62.6/52.8-74	65.5 ± 9.6<br>67.5-61.8-72.4	52.7 ± 16.3<br>57.1/39-65.7	43.4 ± 12.5<br>46.3/33.4-54.7
CD4+ TEMRA<br>(% of CD4+)	3.2 ± 4.6<br>1.1/0.3-3.9	2 ± 3.1<br>0.7/0.3-2.2	5 ± 6.5<br>2.1/0.5-6.4	2.1 ± 1.8<br>2/0.4-3.3
CD8+<br>(% of CD3+)	34.6 ± 10.3<br>33/26.6-42.6	28.5 ± 6.1<br>27.4/24-33.2	29.4 ± 5.9<br>31/23.6-33.8	28.6 ± 7.2<br>26.4/23.9-32.2
CD8+ HLADR<br>(% of CD8+)	7.9 ± 5.7<br>6.1/3.5-11.5	9.8 ± 5<br>9/6.7-12.6	7.3 ± 3.1<br>6.9/4.9-8.7	22.2 ± 12.8<br>20.1/9.5-34.6
CD8+ central memory<br>(% of CD8+)	7.1 ± 4.3<br>5.9/4.4-9	12.1 ± 6.3<br>11.6/7.6-15.9	10.1 ± 5.7<br>8.5/6.9-11.8	7.3 ± 4.4<br>5.4/3.2-10.7
CD8+ effector memory<br>(% of CD8+)	13 ± 6.9<br>11.8/7.4-16.7	16.9 ± 6.3<br>16.8/13-21.7	12.2 ± 5.5<br>10.6/7.7-16.9	15.6 ± 8.5<br>14.5/8.5-23.6
CD8+ effector memory 1<br>(% of CD8+ effector memory)	64.1 ± 13.8<br>64.2/50.9-73.7	72.4 ± 13.8<br>74.7/65.4-83.9	69.7 ± 12.1<br>70.1/62.5-79.1	62.5 ± 24.8<br>67.3/40.4-86.3
CD8+ effector memory 2<br>(% of CD8+ effector memory)	13.6 ± 9.6<br>10.1/6.5-22.3	10.4 ± 9.8<br>8.7/0.7-18.5	6.2 ± 7.8<br>4.5/0.1-9.3	22.3 ± 23.1<br>12.4/2.6-45.8
CD8+ effector memory 3<br>(% of CD8+ effector memory)	10.1 ± 9.2<br>7.3/3.3-13.4	6.5 ± 9.7<br>4.4/0.2-9	6.1 ± 8<br>2.9/0.2-9.4	10.6 ± 17.2<br>3.5/0.5-14.5
CD8+ effector memory 4<br>(% of CD8+ effector memory)	12.1 ± 10.2<br>8.8/4.4-16.3	10.7 ± 4.9<br>10.7/7.1-13.9	18 ± 13.1<br>16.3/8.3-21.4	4.6 ± 3.2<br>5.9/1.3-6.9
CCR7+ CD45RA+<br>(% of CD8+)	33.7 ± 14.9<br>34.3/22.1-44	30.4 ± 11.5<br>29.6/20.4-38.8	28.2 ± 8.8<br>31/18.7-35.4	19.6 ± 11.9<br>18.4/9.2-25.4
CCR7+ CD45RA+ CD31-<br>(% of CD8+ CCR7+ CD45RA+)	4.1 ± 3<br>3.6/2-5	4.8 ± 3.3<br>3.9/2.8-5.3	5.3 ± 2.9<br>4.6/3.2-7.2	9.7 ± 8<br>7.3/3.1-15.6
CCR7+ CD45RA+ CD31+<br>(% of CD8+ CCR7+ CD45RA+)	92.5 ± 6<br>95/89-96.4	91.7 ± 5.7<br>93.9/90.1-95.8	89.7 ± 6.3<br>90.2/86.9-94.4	88.3 ± 8.3<br>90.8/83.2-94.9
CD8+ TEMRA<br>(% of CD8+)	40.3 ± 20.6<br>36.1/22.6-57.3	33.1 ± 9.5<br>31.1/27.8-40.9	43.7 ± 16.7<br>42.9/35.3-54.3	51.8 ± 19.7<br>43.6/35.8-71.3

Supplementary Table 9: B cells mean and median values

Patients group	Contacts	Asymptomatic	Mild	Severe
	mean ± SD<br>median/IQR	mean ± SD<br>median/IQR	mean ± SD<br>median/IQR	mean ± SD<br>median/IQR
B cells<br>(% of lymphocytes)	7.2 ± 2.3<br>7.2/5.3-8.5	8.4 ± 3.7<br>8.3/6.1-9.4	9.1 ± 4.4<br>9.3/5.8-10.6	5.6 ± 6.3<br>2.9/1.4-8
B transitional<br>(% of B cells)	4.6 ± 2.6<br>4.3/2.5-6.2	5.7 ± 3.2<br>4.3/3-9	4.8 ± 2.7<br>4.5/2.7-5.8	3.2 ± 2.5<br>2.8/0.9-5
Naive B cells<br>(% of B cells)	52.3 ± 16.8<br>50/45.4-64.8	55 ± 17.2<br>56.6/49.3-68.9	54.1 ± 12.3<br>55.3/52.7-63.2	52.4 ± 25.1<br>58.4/30.1-73.9
Memory B cells<br>(% of B cells)	40.7 ± 15.4<br>41.3/29.7-47.2	38.7 ± 16.4<br>35.7/25.3-46.3	39.8 ± 10.2<br>37.8/32.4-41.2	32.4 ± 19.6<br>32/15.8-52.1
Atypical memory B cells<br>(% of B cells)	3.1 ± 2.1<br>2.8/1.3-4.4	2.7 ± 2<br>2.4/1.1-4.1	3.2 ± 4.4<br>2/0.7-3	4.5 ± 4.2<br>2.3/1.5-8.7
Plasmablasts<br>(% of B cells)	2.1 ± 3<br>1/0.6-2.4	1.9 ± 1.3<br>1.7/0.8-2.9	1.8 ± 1.5<br>1.8/0.5-2.2	8 ± 7.9<br>5.3/3.3-10.8
IgM memory B cells<br>(% of memory B cells)	49.8 ± 14.8<br>46.4/41.2-57.3	54.1 ± 17.3<br>56.9/42.4-65.9	61.1 ± 14<br>59.5/52.3-71.4	37.7 ± 19<br>35.5/19.3-54.4
Switched memory B cells<br>(% of memory B cells)	48.7 ± 15.2<br>52.6/40.8-58	43.5 ± 17.7<br>40/30.8-56.3	37.3 ± 14.3<br>39.3/27-46.3	60 ± 18.9<br>61.1/44.4-78.3
IgG memory B cells<br>(% of memory B cells)	22.7 ± 8.9<br>23.6/15.8-29.6	16.7 ± 10.1<br>14.5/11.8-20.1	12.9 ± 6<br>13.9/6.9-18.5	19.9 ± 6.8<br>18/14.2-27.7
IgG- IgM- memory B cells<br>(% of memory B cells)	21.8 ± 8.2<br>22.3/16.8-26	19.5 ± 9.4<br>16.1/12.2-26.5	20 ± 6.3<br>19.6/16.1-24.2	32.3 ± 10.2<br>31.5/26.8-42.3

Supplementary Table 10: B cells total mean and median values

Patients group	Contacts	Asymptomatic	Mild	Severe
	mean ± SD<br>median/IQR	mean ± SD<br>median/IQR	mean ± SD<br>median/IQR	mean ± SD<br>median/IQR
B cells<br>(% of lymphocytes)	7.1 ± 2.3<br>7.2/5.3-8.3	8.7 ± 2.4<br>8.6/7.3-9.8	8.6 ± 4.2<br>8.4/5.9-10.5	6.3 ± 5.4<br>6.1/2.2-8.3
B transitional<br>(% of B cells)	3.6 ± 2.6<br>3.5/1-5.2	6.2 ± 4.7<br>4.9/2.6-9.2	4.8 ± 3.6<br>4.3/1.8-6.2	2.5 ± 1.9<br>1.7/1-4.2
Naive B cells<br>(% of B cells)	41.1 ± 14<br>40.9/31.3-49.8	53.4 ± 17.2<br>54/44.2-66.4	44.5 ± 15.2<br>49.5/33.1-55.1	48.7 ± 21.8<br>54.4/33.8-64.4
Memory B cells<br>(% of B cells)	35.4 ± 12<br>35.6/26.7-41.1	28.7 ± 16<br>24.9/18.9-33.3	36.4 ± 8<br>36.2/30.5-39.4	30.8 ± 17.2<br>27.3/15.6-42
Atypical memory B cells<br>(% of B cells)	3.6 ± 2.1<br>3.4/1.8-5.2	2.6 ± 2<br>1.9/1.2-3.4	3.1 ± 2.5<br>2.3/1.4-3.9	3.2 ± 2.5<br>2.3/1.6-4.8
Plasmablasts<br>(% of B cells)	1.9 ± 2.4<br>1/0.6-2.2	1.5 ± 0.9<br>1.3/0.9-1.8	1.6 ± 1.1<br>1.1/0.9-1.9	5.9 ± 4.7<br>4.6/3-6.8
IgM memory B cells<br>(% of memory B cells)	47.6 ± 10.2<br>47.3/40.5-53.6	48.4 ± 11.5<br>49.8/39-55.4	61.4 ± 12.6<br>64.3/50.2-69.8	44.2 ± 20.5<br>47/22.8-64.5
Switched memory B cells<br>(% of memory B cells)	44.6 ± 14.4<br>46.6/36.6-54.1	43.2 ± 13.6<br>42.4/33.4-52.7	33.2 ± 13.6<br>33.9/25.4-44.5	53 ± 21.4<br>51.3/32.7-75
IgG memory B cells<br>(% of memory B cells)	19.3 ± 8.4<br>18.4/15.1-24.1	17.1 ± 7.6<br>17.3/11-22.7	10.7 ± 6.7<br>10.3/5.8-14.8	15.3 ± 8.8<br>14.9/8.8-22.1
IgG- IgM- memory B cells<br>(% of memory B cells)	22.7 ± 9.5<br>21.7/17.4-27.4	23 ± 10.8<br>21.6/15.3-28.2	20.4 ± 8.6<br>20.6/15.3-26	34.6 ± 15.7<br>32.4/23.7-43.2
